# The Treatment of Syphilitic Affections

**Published:** 1845-07

**Authors:** 


*The treatment of Syphilitic Affections.*—At a recent meeting of the Surgical Society of Ireland, the proceedings of which are reported in the ***Dublin Medical Press***, an interesting paper was read by Dr. M'Egan, on the diagnosis and treatment of syphilitic diseases. In the discussion to which this paper gave rise, the president, Mr. Carmichael took occasion to give a brief account of the origin and progress of his own investigations on the subject, and after alluding to the opposition which his views had at first encountered, and exposing the attempts which had been made to suppress them, by some practices of very questionable character, concluded by making the following observations in reference to the employment of mercury.

1st. I do not think it necessary in the treatment of the simple primary ulcer without induration, nor for the papular eruption, and other constitutional symptoms it produces; but should the eruption linger into the fourth or fifth week after it has desquamated into scaly spots or blotches, mercury in alterative doses, either in the form of Plummer's pill or the proto-ioduret of mercury, will be of service in clearing the skin of the eruption, and in removing the pains of the joints, which are constantly present in this form of venereal. But I protest most strongly against the use of mercury at the period when the eruption first appears in its papular form, at a time that it is usually preceded and accompanied by considerable fever, like all the other exanthemata, to which class of Cullen it obviously belongs. If we should exhibit mercury prematurely during the eruptive stage of this as well as the other forms of disease, the scaly excepted, we may possibly clear the skin of the eruption, but in all probability it will return again and again, to the great disappointment of the patient and perplexity of the medical attendant.

2nd. For iritis I would give mercury, so as to excite its full effect upon the system, and at the same time not neglect the usual antiphlogistic measures to remove this dangerous inflammation.

3rd. For nodes I would exhibit mercury, and I think the iodide of that mineral for their removal is superior to any other preparation.

4th. For the phagedenic primary ulcers I have always found mercury most injurious. They are most successfully treated by the application of strong nitric acid, immediately followed by a douche of cold water. The same application is also the most efficient for phagedenic ulceration of the throat, which if not checked will soon extend over the velum, uvula, and back of the pharynx, from whence it will spread upwards into the nares, and downwards into the larynx; in either of which situations I need not state the difficulty and danger of the case. Instead of the douche of cold water, in this situation inadmissible, I employ a probang; the sponge moistened in a solution of soda or potash will neutralize any superabundant acid applied to the ulcers. During the eruption of pustules or tubercles which cause those crusts termed rupia, I have found mercury injurious, although its exhibition may at first flatter both patient and surgeon that the disease is yielding to this remedy. But the natural tendency of this eruption is also to become scaly after it has existed several weeks or months. This scaliness is a sign that the disease is on the decline, and indicates that mercury in alterative doses may then be employed with safety and advantage. Should any of the constitutional ulcers on the skin spread after the rupia crusts fall off, their progress may also be effectually checked by the application of nitric acid to their phagedenic margins. They of themselves first show signs of healthy reparation in their centres, which need not therefore be meddled with. Mercury in this stage of the disease should not be exhibited. Hydriodate of potash, sarsaparilla, country air, and the tranquillizing effects of opium, should the patient be harassed by extensive ulceration, are the constitutional means most to be relied on.

5th. For the true Hunterian chancre with hardened edge and base, and for the scaly eruption which attends it, as well as the deep excavated ulcer of the tonsil, nodes, and other symptoms belonging to this form of disease, mercury may be esteemed a certain and expeditious remedy.

These are the views I entertained more than thirty years ago, as appear from my various publications on the subject; and it surprises even myself not a little that after so long experience, I have felt no occasion or necessity to depart from them. But I have often been pained at finding that many of the profession, particularly in the army, range themselves under the denomination of mercurialists or anti-mercurialists—*i. e.*, they either exhibit mercury in all venereal cases or refrain from it in every instance, regardless of the characters of the symptoms and stages of the disease. It may, however, be satisfactory to those who are indolently disposed, and wish to follow routine, to know that mercury may be given in general with advantage when an eruption is *scaly*—no matter whether or not it has been scaly from the commencement.

In fine, from my publications, as well as from what I have stated this night, it is obvious, that although in the great majority of cases I consider the exhibition of mercury unnecessary or injurious, yet in one form of disease, that which produces the scaly eruption, lepra or psoriasis, it is the remedy upon which I place my sole reliance; and in other forms of venereal which produce the papular, pustular, tubercular, or rupia eruptions, there are stages and states in which it will be found a most useful auxiliary. Therefore, though it may accord with the natural indolence of our dispositions to treat all cases in either one way or the other, yet no practitioner, I aver, will be successful in following this routine mode of practice, who does not exert his powers of discrimination to ascertain to which class the symptoms under treatment belong; for no matter, in a practical point of view, whether there is one or a plurality of venereal poisons, (as I advocate,) it is of the utmost consequence, to ensure an efficient and successful issue, to consider the form of the disease under consideration, and to which of my classes it appertains.—*Ibid.*

